# The Contributing Factors of Delayed-Onset Post-traumatic Stress Disorder Symptoms: A Nested Case-Control Study Conducted After the 2008 Wenchuan Earthquake

**DOI:** 10.3389/fpubh.2021.682714

**Published:** 2021-12-24

**Authors:** Yanlin Yang, Wenqi Zeng, Bingqing Lu, Jin Wen

**Affiliations:** ^1^Institute of Hospital Management, West China Hospital, Sichuan University, Chengdu, China; ^2^Hospital of Chengdu University of Traditional Chinese Medicine, Chengdu, China

**Keywords:** earthquake, delayed-onset PTSD, contributing factors, mental health, public health policy

## Abstract

**Background:** Delayed-onset post-traumatic stress disorder after catastrophes is a major public health issue. However, good designs for identifying post-traumatic stress disorder (PTSD) among earthquake survivors are rare. This is the first nested case-control study to explore the possible factors associated with delayed-onset PTSD symptoms.

**Methods:** A nested case-control study was conducted. The baseline (2011) and follow-up (2018) surveys were utilized to collect data. A total of 361 survivors of the Wenchuan earthquake were investigated and 340 survivors underwent follow-up. The survivors, from the hardest-hit areas, who met the criteria for PTSD were included in the case group, and PTSD-free survivors from the same area, matched for age, were included in the control group, with a ratio of one to four. Conditional logistic regression was used to evaluate the variables' odds ratio (OR).

**Results:** The overall prevalence of delayed-onset PTSD symptoms in survivors of the Wenchuan earthquake was 9.7% (33/340). The unemployed earthquake survivors had a higher risk of developing delayed-onset PTSD symptoms (OR = 4.731, 95% CI = 1.408–15.901), while higher perceived social support was a protective factor against delayed-onset PTSD symptoms (OR = 0.172, 95% CI = 0.052–0.568).

**Conclusion:** Delayed-onset PTSD symptoms, after a disaster, should not be ignored. Active social support and the provision of stable jobs can contribute to the earthquake survivors' mental health.

## Introduction

Since earthquakes are not always predictable and are highly destructive, they can cause significant damage to physical and mental health (1). Moreover, since 1950, earthquakes have become the most devastating natural disaster (2). The Wenchuan earthquake, which occurred in Sichuan province on May 12, 2008, and resulted in 69,227 deaths, 374,643 injuries, and 17,923 people missing, was the most ruinous earthquake since its founding of the People's Republic of China (3, 4). Furthermore, the economic losses reached 84.51 billion Renminbi. In addition, it placed a great psychological burden on the survivors (5). A study of 2080 Wenchuan earthquake survivors found that 40.1% of the participants suffered from post-traumatic stress disorder (PTSD) 1 year after the earthquake (6). Similarly, a related meta-analysis report showed a 29% incidence of PTSD in a sample of 76,101 survivors in the 9 months following the earthquake (7).

Post-traumatic stress disorder, a severe and complex mental disorder caused by exposure to a catastrophic event, is composed of three clusters of symptoms: re-experiencing, avoidance, and hyperarousal (8). PTSD onset can be close to the traumatic event or delayed (9), and some populations will never present with PTSD despite similar traumatic exposure (10, 11), so the symptoms of PTSD after the disasters could take multiple trajectories (12). One study revealed four trajectories of PTSD: resilience, recovery, chronic, and delayed (13, 14). Compared to it, a population-based longitudinal study identified six clusters of PTSD symptom trajectories after the disaster: low-stable, moderate-stable, moderate-increasing, high-stable, high-decreasing, and very high-stable (15).

About 70% of the world's ordinary people will experience potential traumatic events (PTE) in their lifetime (16), and many people will have post-traumatic stress symptoms (PTSS) (17). People with PTSS are at a higher risk of developing delayed-onset PTSD, especially after experiencing subsequent PETs or other stressors (18). Unfortunately, both PTSS survivors and PTSD survivors may have their brain function and structure changed after trauma, and trauma survivors are at high risk of developing mental disorders. For example, research has shown that the appearance of post-traumatic nightmare indicated delayed-onset PTSD, even if the delayed-onset PTSD has been solved, the nightmare associated with the PTSD would persist throughout life (19). Another study also confirmed that lifetime PTSD affected about 10% of women and 5% of men in the general population (20, 21). Moreover, a study focused on the nervous system and revealed the survivors' cognitive might decline, which might lead to loss of well-being in later life (22, 23). In addition, according to the stress sensitization hypothesis, that individuals who have experienced previous PTSD are more susceptible to developing PTSD following subsequent traumas (24, 25). In a word, our research on delayed-onset PTSD has a lot of scientific implications.

In delayed-onset PTSD, the PTSD symptoms are initially at a low level but increase gradually over time (13). Additionally, a study found that a significant number of survivors developed PTSD after six or more months after the initial traumatic event (26). Studies have demonstrated that people continued to suffer from PTSD 8 years after the earthquake (27–32). These studies focused on factors related to PTSD, such as alexithymia (27), post-traumatic growth (28), self-esteem (29), depression (30), suicidal behaviors (31), and community support (32). However, all of them were cross-sectional studies and none of them focused on delayed-onset PTSD. After reviewing a large number of studies, perceived social support was considered an important variable among the various factors affecting PTSD (33–36). Moreover, according to the stress vulnerability model (37), emotional problems under stress are related to the individual's vulnerability to stress, the magnitude of stress, the impact of the environment, and the ability to cope with stress (38). PTSD is trauma and stress-related disorder (39), therefore, predictor variables in this study were selected based upon 3 levels: (1) individual factors, (2) social factors, and (3) disaster-related factors. We aimed to explore the relationship between these factors and delayed-onset PTSD symptoms by conducting a nested case-control study.

## Methods

### Ethics Statement

This study was approved by the institutional review board of the West China Hospital of Sichuan University. The purpose and significance of the study were described in detail, and oral informed consent was obtained from each participant prior to the survey.

### Study Design and Setting

This was an observational study using the nested case-control methods. The first survey was conducted in Wenchuan, Shifang, and Mianzhu—areas that were affected by the earthquake—from May to June 2011, and the follow-up survey was conducted from April to October 2018.

### Cases and Controls

We used the PTSD Checklist-Civilian Version (PCL-C) scale, which was based on the fourth edition of the Diagnostic and Statistical Manual of Mental Disorders, to screen for PTSD symptoms related to earthquakes (40). Both English and Chinese versions of PCL-C were often used when a clinical interview was not feasible and had been demonstrated to have good validity, reliability, and accuracy in screening PTSD (41). In a previous study, the Chinese version of the PCL-C was validated and showed good internal reliability (Cronbach's alpha = 0.89) (40). This self-report scale contained 17 items and its total score ranged from 17 to 85 (42).

It was worth noting that we did not diagnose PTSD but screened for PTSD among this sample thus measuring PTSD symptomatology rather than PTSD. Survivors who were not screened for PTSD symptoms during the first survey and those with a score of 38 and above during the follow-up survey were classified as likely to have delayed-onset PTSD symptoms and were included in the case group. The control group comprised survivors, from the same area, with a PCL-C score <38, with a ratio of 1:4, and were matched for age (±2years).

### Variables and Measurements

Demographic information (age, gender, nationality, education, and profession) and other factors (smoking behavior, drinking behavior, the prevalence of chronic diseases, whether injured during the earthquake, having relatives who were injured, disabled or killed, due to or during the earthquake) were collected using a predefined questionnaire. Participants with a formal diagnosis of at least one of the following were considered to have chronic diseases: malignant tumor, heart disease, chronic non-specific lung disease (asthma, bronchitis, and emphysema), atherosclerotic disease, cerebrovascular disease (stroke, excluding transient ischemic attack), diabetes, osteoarthritis, and rheumatoid arthritis. Family members who were injured, disabled, or killed in the earthquake were classified into two groups based on their relationship to the participants: the first group included parents, spouses, and children, and the second included grandparents, grandchildren, siblings, and other relatives.

Perceived social support was tested using the Perceived Social Support Scale (PSSS), which was considered to be reliable to measure social support (43), and the Chinese version of the PSSS was validated and showed good internal reliability (Cronbach's alpha = 0.89) in previous studies (44, 45). This self-report scale, which measured the support from three aspects—family, friends, and others—contained 12 items and its total score ranged from 12 to 84, with a higher score indicating a higher degree of perceived social support (46). A total score below 50 was defined as a low degree of perceived social support, and a score of 50 and above was defined as a high degree of perceived social support (47, 48).

### Bias

A multistage random sampling procedure was performed to select the study sample to reduce selection bias. The field interviewers included both clinical psychologists and graduate students from preventive and clinical medicine, and all of them received uniform training prior to the commencement of the survey. Survivors with any pre-existing mental disorder were excluded at the baseline. Double entry of the questionnaire data was independently carried out by two trained staff for validation and quality assurance. Multivariate logistic regression analysis was used to adjust for potential confounding factors.

### Statistical Analysis

Statistical analyses were performed using SPSS version 25 (IBM Corp. in Armonk, New York, U.S.). Categorical data were using frequencies and percentages. Means and standard deviations were analyzed for continuous variables and conditional logic regression was used to identify the risk factors for delayed-onset PTSD symptoms. The odds ratios (ORs) and 95 % CIs both were also calculated.

## Results

**Figure 1:**
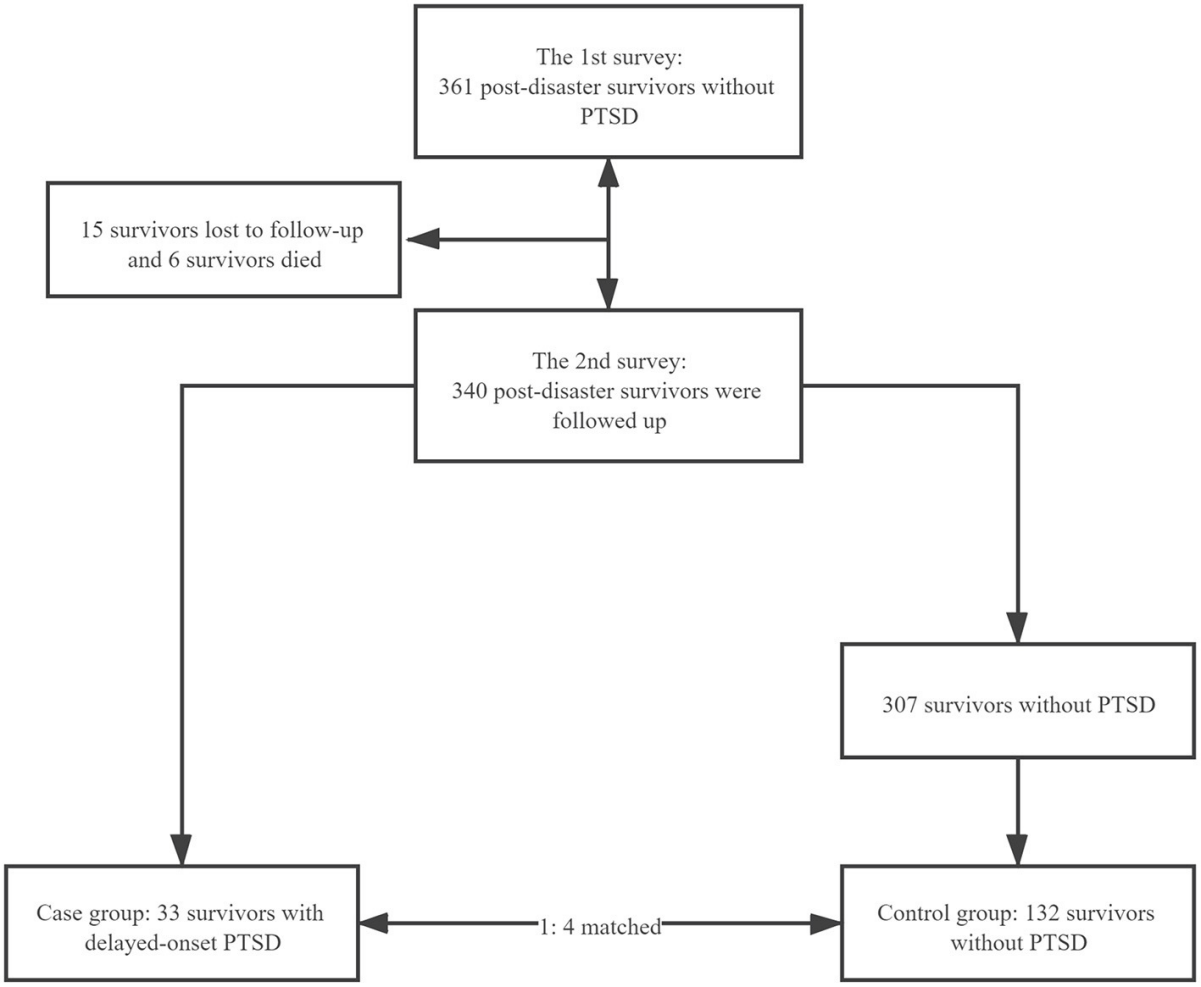
Flowchart of sampling.

A total of 361 survivors without PTSD symptoms from the first survey were included in the cohort. During follow-up, it was found that the prevalence of delayed-onset PTSD symptoms was 9.7% (33/340). A considerable proportion of traumatized individuals subsequently developed PTSD. A meta-analysis showed that 27.0% of individuals developed initial PTSD symptoms but then recovered, 10.3% developed chronic and 6.4% had delayed-onset PTSD (14, 49). This supported the prevalence of delayed-onset PTSD symptoms in our study to some extent. Meanwhile, 33 cases were identified, and 132 controls were matched with these cases (Figure 1). The mean age was 58.03 and 58.85 in the case and control groups, respectively. The majority of those in the case group were female, were a minority, were illiterate, had primary school education, were unemployed, were other professionals except farmers, workers, and unemployed people, did not smoke, did not drink, had chronic diseases, were either injured themselves or had a family member who was injured, disabled, or killed by the earthquake, and had lower perceived social support than those in the control group (Table 1).

**Table 1:** Demographic characteristics of cases and matched controls.

**Characteristic**	**At baseline, No. (%)**	**During follow-up, No. (%)**	
		**Case** **(*n* = 33)**	**Control** **(*n* = 132)**
Age, years (mean, SD)	50.88 (9.93)	58.03 (10.04)	58.85 (9.94)
Gender			
Male	61 (37.0)	10 (30.3)	51 (38.6)
Female	104 (63.0)	23 (69.7)	81 (61.4)
Nation			
Minority	29 (17.6)	9 (27.3)	20 (15.2)
Han ethnic	136 (82.4)	24 (72.7)	112 (84.8)
Education			
Illiteracy	53 (32.1)	13 (39.4)	40 (30.3)
Primary school	71 (43.0)	15 (45.5)	56 (42.4)
Junior high school or above	41 (24.8)	5 (15.2)	36 (27.3)
Profession			
Farmer	51 (30.9)	7 (21.2)	48 (36.4)
Worker	49 (29.7)	5 (15.2)	37 (28.0)
Unemployed	57 (34.5)	19 (57.6)	41 (31.1)
Others	8 (4.8)	2 (6.1)	6 (4.5)
Smoking			
No	134 (81.2)	29 (87.9)	107 (81.8)
Yes	31 (18.8)	4 (12.1)	25 (18.9)
Drinking			
No	123 (74.5)	27 (81.2)	92 (69.7)
Yes	42 (25.5)	6 (18.2)	40 (30.3)
Chronic disease			
No	62 (37.6)	6 (18.2)	34 (25.8)
Yes	103 (62.4)	27 (81.2)	98 (74.2)
Injured in earthquake			
No	137 (83.0)	25 (75.8)	112 (84.8)
Yes	28 (17.0)	8 (24.2)	20 (15.2)
Family member injured during earthquake			
No	105 (63.6)	6 (54.5)	87 (65.9)
Yes	60 (36.4)	27 (45.5)	45 (34.1)
Family member disabled during earthquake			
No	154 (93.3)	27 (81.8)	127 (96.2)
Yes	11 (6.7)	6 (18.2)	5 (3.8)
Family member died during earthquake			
No	86 (52.1)	17 (51.5)	69 (52.3)
Yes	79 (47.9)	16 (48.5)	63 (47.7)
PSSS			
Low	32 (19.4)	7 (21.2)	18 (13.6)
High	133 (80.6)	26 (78.8)	114 (86.4)

In the univariate analysis, unemployment increased the risk of developing delayed-onset PTSD symptoms (crude OR = 2.987, *P* < 0.05); those with family members who were disabled during the earthquake were also at risk of developing delayed-onset PTSD symptoms (crude OR = 4.8, 95% CI = 1.465–15.728). However, a higher degree of perceived social support was a protective factor against developing PTSD symptoms (crude OR = 0.201, 95% CI = 0.082–0.493) (Table 2).

**Table 2:** Results of conditional logistic regression analyses.

	**Univariate estimate**			**Multivariate estimate**		
	**OR**	**95% CI**	***P***	**OR**	**95% CI**	***P***
Gender						
Male	Ref.			Ref.		
Female	1.464	0.636–3.371	0.371	0.672	0.179–2.524	0.556
Nation						
Minority	Ref.			Ref.		
Han ethnic	0.421	0.154–1.150	0.091	0.637	0.173–2.351	0.499
Education						
Illiteracy	Ref.			Ref.		
Primary school	0.716	0.281–1.823	0.484	0.968	0.261–3.592	0.962
Junior high school or above	0.36	0.103–1.259	0.11	0.245	0.038–1.584	0.14
Profession						
Farmer	Ref.			Ref.		
Worker	0.829	0.229–3.009	0.776	0.693	0.125–3.853	0.675
Unemployed	2.987	1.147–7.774	0.025	4.731	1.408–15.901	0.012
Others	2.237	0.401–12.490	0.359	3.847	0.431–34.364	0.228
Smoking						
No	Ref.			Ref.		
Yes	0.597	0.195–1.833	0.368	0.816	0.129–5.175	0.829
Drinking						
No	Ref.			Ref.		
Yes	0.524	0.204–1.345	0.179	0.734	0.137–3.939	0.719
Chronic disease						
No	Ref.			Ref.		
Yes	1.58	0.593–4.213	0.36	1.908	0.524–6.943	0.327
Injured in earthquake						
No	Ref.			Ref.		
Yes	1.762	0.712–4.361	0.221	3.138	0.911–10.81	0.07
Family member injured during earthquake						
No	Ref.			Ref.		
Yes	1.639	0.747–3.596	0.217	2.068	0.642–6.666	0.224
Family member disabled during earthquake						
No	Ref.			Ref.		
Yes	4.8	1.465–15.728	0.011	2.364	0.409–13.668	0.336
Family member died during earthquake						
No	Ref.			Ref.		
Yes	1.029	0.492–2.152	0.94	1.332	0.469–3.781	0.59
PSSS						
Low	Ref.			Ref.		
High	0.201	0.082–0.493	<0.001	0.172	0.052–0.568	0.004

A multivariable conditional logistic regression analysis was then performed. However, after adjusting for the other variables, the family members who were disabled during the earthquake variable demonstrated no statistical significance. In the multivariate analysis, unemployment and a higher degree of perceived social support were also important factors in developing delayed-onset PTSD symptoms. Unemployment was a risk factor for delayed-onset PTSD symptoms (adjusted OR = 4.731, 95% CI = 1.408–15.901), while a higher degree of perceived social support was a protective factor (adjusted OR = 0.172, 95% CI = 0.052–0.568).

## Discussion

The aim of this nested case-control design was to identify the influence of latent factors—personal, social, and disaster-related variables—on delayed-onset PTSD symptoms. The findings revealed that unemployment increased the risk of delayed-onset PTSD symptoms, while a higher degree of perceived social support reduced the risk of PTSD symptoms.

It was necessary to point that we assessed PTSD among survivors at the follow-up survey 10 years after the Wenchuan earthquake so that this elapsed time might have also influenced the delayed-onset PTSD system. According to the stress vulnerability model, that the course of severe mental illness was determined by an interaction of biological vulnerability, stress, and coping (50). Therefore, other subsequent traumas (even of minor severity as grief) could act by adding their burden to promote the development of PTSD (51). To control for bias, we emphasized to the subjects that this was an investigation into the impact of the Wenchuan earthquake in 2008 during our follow-up, and when the investigators asked the subjects questions, they would repeatedly emphasize the words Wenchuan earthquake, in an attempt to guarantee that delayed-onset PTSD was promoted by the Wenchuan earthquake.

Though those who were unemployed and retired were both not actively employed, only those who were unemployed were vulnerable to PTSD (52); since those who were unemployed did not have a stable income, however, most of the retirees had pensions to cover their living expenses. For unemployed post-earthquake, evidence showed that loss of own resources such as income (53, 54), was the most influential exposure variable for mental illness. This indicates that income is inherently a crucial factor in developing delayed-onset PTSD (40), therefore, the local government should create and provide more jobs to help the survivors guarantee adequate income.

Both social and interpersonal factors played a vital role in the trauma recovery process (55); we also found that a high degree of perceived social support could reduce the occurrence of delayed-onset PTSD symptoms. Furthermore, it should be emphasized that actual social support was not equal to perceived social support, and existing literature has demonstrated that perceived social support was more beneficial for survivors, to adapt and cope with stress after catastrophes (35). Therefore, we should provide practical and acceptable help for survivors through more effective communication with them, not just by providing routine social support.

A previous study found that there was a negative correlation between disability due to natural disasters and PTSD (56). As shown in the univariate analysis, disability also increased the risk of delayed-onset PTSD symptoms due to the presence of family members with disabilities. This could be because caring for relatives with disabilities amplifies the personal suffering experienced during the earthquake, and some studies also showed that severe illnesses in family members might represent a complex burden for their caregivers, including a wide range of mental disorders, particularly PTSD (57, 58). For caregivers of disabled family members after the Wenchuan earthquake, they often had to struggle to adjust to new responsibilities and roles and were faced with double stressors-the stress of evoked memories about the Wenchuan earthquake and the burden of family care, so they had the risk for development of delayed-onset PTSD symptoms. Nevertheless, the multivariate analysis revealed an insignificant relationship between family members' disability and delayed-onset PTSD symptoms, which might be because family members' disability was not the most significant factor for developing delayed-onset PTSD symptoms, compared with unemployment and social support.

Three previous studies showed that subthreshold PTSD was associated with an increased risk of suicidality (59, 60, 64). Hence, taking into account survivors with both partial and sub-threshold PTSD symptoms was also important. Some studies focused on the survivors with subthreshold manifestations in a dimensional perspective. For example, if the survivors were defined as a report of at least one symptom in Criteria B (re-experiencing), C (avoidance), and D (hyperarousal), they would be considered as having “Partial PTSD” (61). Another study used The Harvard Trauma Questionnaire part IV (HTQ) to measure the presence of PTSD, patients were given a possible PTSD diagnosis if they reported at least one re-experiencing symptom, three avoidance symptoms, and two hyper-arousal symptoms (62).

Moreover, the definition of the related post-traumatic stress symptoms is a hot topic of discussion in the recent past. For example, the DSM-V shifted from the previous three symptomatological criteria in DSM-IV to a four-criteria structure: Intrusion symptoms; Persistent avoidance; Negative alterations in cognitions and mood; Alterations in arousal and activity (51). Trauma and Loss Spectrum-Self Report (TALS-SR), which represented a valuable tool to assess the spectrum of clinical manifestations related to DSM mental disorders, also explored post-traumatic stress spectrum symptoms related to the three symptomatic criteria for PTSD diagnosis that were provided by the DSM-IV in its' Domains V, VI and VIII. The TALS-SR has been used to investigate both full and partial PTSD among survivors after L'Aquila 2009 Earthquake (63). These findings are important for the planning of future research since we can make a comprehensive evaluation of the PTSD symptoms.

This study has several strengths. First, to the best of our knowledge, this is the first nested case-control study, which is known for revealing the relationship between possible factors and delayed-onset PTSD symptoms after an earthquake. Second, this is a decade-long follow-up study, and the demonstrated relationships between unemployment/social support and delayed-onset PTSD symptoms are critical for local governments to promote survivors' health, which may be needed even 10 years after a catastrophe.

There are some limitations to this study. First, since the PTSD symptoms were not formally diagnosed by psychiatrists, self-report biases might exist. Second, the sample size was small, but the main reason for this was that most of the investigated survivors inhabited the mountainous regions, and the investigation was therefore difficult. Third, psychopathological comorbidities like mood disorders, anxiety disorders, or alcohol and substance abuse might have influenced the presence of PTSD symptoms at the second evaluation 10 years after the event (59). In addition, although we found some significant factors related to PTSD symptoms, our data were unable to shed light on causal associations.

Finally, further studies which are based upon clinician diagnosis would be needed in the future and using the Clinician-Administered PTSD Scale (CAPS) which is considered the “gold standard” for measuring PTSD is necessary (60). Moreover, the exact causative relation needs further exploration.

## Conclusion

In summary, the current evidence demonstrates that delayed-onset PTSD symptoms continue to affect earthquake survivors; fortunately, a higher degree of perceived social support would reduce the risk of delayed-onset PTSD symptoms. However, it should be noted that unemployment increases the likelihood of delayed-onset PTSD symptoms. Therefore, social support should be provided not only through psychological counseling but also through targeted unemployment assistance to help survivors improve their quality of life.

## Data Availability Statement

The datasets presented in this article are not readily available because the data supporting the findings of the article are not publicly available, but it can be provided by the corresponding author on reasonable request. Requests to access the datasets should be directed to Jin Wen, huaxiwenjin@163.com.

## Ethics Statement

The studies involving human participants were reviewed and approved by the Institutional Review Board of West China Hospital of Sichuan University. The patients/participants provided their written informed consent to participate in this study. The ID of the ethical consent form is 2019343.

## Author Contributions

YY, WZ, BL, and JW conceived the idea for the study and designed the study methodology. YY and WZ accessed and validated the dataset, did the formal data analysis, and prepared the first draft of the manuscript. YY and BL curated the data. JW supervised the study and acquired the funding for the study. All authors agreed to be cited as co-authors, accepting the order of authorship, and approved the final version of the manuscript and the manuscript submission to Frontiers in Public Health, also did the investigation, participated in the critical revision of the manuscript, and were responsible for the figures.

## Funding

This work was supported by the National Natural Science Foundation of China (grant number 71874115).

## Conflict of Interest

The authors declare that the research was conducted in the absence of any commercial or financial relationships that could be construed as a potential conflict of interest.

## Publisher's Note

All claims expressed in this article are solely those of the authors and do not necessarily represent those of their affiliated organizations, or those of the publisher, the editors and the reviewers. Any product that may be evaluated in this article, or claim that may be made by its manufacturer, is not guaranteed or endorsed by the publisher.

## Abbreviations

PTSD: post-traumatic stress disorder
PCL-C: PTSD Checklist-Civilian
PSSS: perceived social support scale
OR: odds ratio
CI: confidence interval.
